# DNA-Loaded Extracellular Vesicles in Liquid Biopsy: Tiny Players With Big Potential?

**DOI:** 10.3389/fcell.2020.622579

**Published:** 2021-01-21

**Authors:** Susana García-Silva, Miguel Gallardo, Héctor Peinado

**Affiliations:** ^1^Microenvironment and Metastasis Laboratory, Molecular Oncology Programme, Spanish National Cancer Research Center (CNIO), Madrid, Spain; ^2^H12O - CNIO Hematological Malignancies Clinical Research Unit, Clinical Research Programme, Spanish National Cancer Research Center (CNIO), Madrid, Spain

**Keywords:** extracellular vesicles, exosomes, cancer, liquid biopsy, plasma, cfDNA

## Introduction

Liquid biopsy in cancer is a revolutionary diagnostic concept defined by the analysis of biological material of tumor origin that extravasate to body fluids. Most common liquid biopsy studies use circulating tumor cells (CTCs) or circulating tumor-derived factors, in particular, circulating tumor DNA (ctDNA) (Alix-Panabières and Pantel, 2016; Perakis and Speicher, 2017). In the last 5 years, cutting-edge technologies such as next-generation sequencing (NGS) or digital PCR (dPCR) have been applied to detect blood-based, tumor-specific biomarkers such as CTCs and ctDNA (Husain and Velculescu, 2017; Pantel and Alix-Panabières, 2019). The quantification of circulating DNA molecules or CTCs showed *per se* prognostic value in many cancers (Haber and Velculescu, 2014). Besides this immediate analysis, another advantage of these biomarkers resides in the possibility of testing specific mutations, methylation profiles, and other DNA patterns (cfDNA and CTCs) and alternatively, proteins and the possibility of generating patient-derived xenografts (PDX) from the most aggressive cells in the tumor that putatively could initiate metastatic outgrowth (CTCs). Furthermore, the development of high sensitivity and specificity techniques enabled the identification of minimal residual disease (MRD) in cancer patient's follow-up blood samples (Pantel and Alix-Panabières, 2019). Complementary to these biomarkers, extracellular vesicles (EVs) are emerging as powerful biomarkers to provide information about the tumor and the systemic changes occurring during the disease. EVs are a broad and heterogenous group of vesicles secreted by almost any kind of cell that display a wide range of sizes (30 nm−5 μm in diameter Witwer and Théry, 2019) and are composed of a lipid bilayer enclosing nucleic acids, proteins, lipids, metabolites (Colombo et al., 2014). EVs are considered as a mechanism of cell-cell communication regulating paracrine and distal cell communication (Tkach and Théry, 2016). According to this, EVs have been detected in most biological fluids (Wiklander et al., 2019). The isolation of EVs allows for the subsequent analysis of their content that is defined by the cell of origin of the vesicle. Due to their heterogeneous content (protein, nucleic acids, lipids, metabolites, etc.), their ubiquitous production by body cells and detection in most biological fluids, circulating EVs could be useful for specific or multiplatform analyses to provide an accurate evaluation of cancer disease at early time points, during progression, therapy and post-treatment facilitating the detection of minimal residual disease and relapse anticipation (LeBleu and Kalluri, 2020).

## EV-Shed DNA, Characteristics and Considerations

Since 2010, several studies have shown the presence of different DNA species in EVs such as ssDNA (Balaj et al., 2011), dsDNA (Kahlert et al., 2014; Thakur et al., 2014) and mtDNA (Cai et al., 2016; Lötvall et al., 2016). Although it is widely accepted that EVs carry DNA, the origins, localization and biological properties of this DNA are not entirely understood (Malkin and Bratman, 2020). Some works support that DNA secretion in EVs is an integral mechanism of clearing cytosolic DNA to maintain cellular homeostasis and avoid senescence and apoptosis (Takahashi et al., 2017). Another report suggests that DNA is a danger-associated signal secreted by dying cells promoting inflammation and anti-tumor immune responses (Kitai et al., 2017). A role for EV-associated DNA in priming and conferring protection to dendritic cells against pathogen infection has also been described (Torralba et al., 2018). Regarding the mechanism involved in DNA secretion on EVs, although microvesicles and exosomes have been proposed to carry DNA, a detailed analysis of the involved mechanism is missing. In a landmark study, Coffey and colleagues found that small EVs did not contain detectable amounts of dsDNA (Jeppesen et al., 2019). Surprisingly, they found that dsDNA was associated with non-vesicular particles that can be co-isolated in the same fraction than small EVs.

Regardless of their origin and mechanism, it appears clear that most of the EV-shed DNA is located on the outside surface of the EVs (Lazaro-Ibanez et al., 2019). However, after DNAse treatment, still a small proportion of DNA remains inside the vesicles (Thakur et al., 2014; Lazaro-Ibanez et al., 2019). Surface-associated DNA confers increased zeta-potential to vesicles, favors exosome-cell adhesion and contributes to the vesicle internalization in the target cell (Fischer et al., 2016; Lötvall et al., 2016; Németh et al., 2017).

Interestingly, all chromosomes are represented in EV-shed DNA with no specific regions overrepresented (Lazaro-Ibanez et al., 2019) suggesting the absence of a sequence-specific loading of DNA species in vesicles. Despite the lower amount of EV-enclosed DNA compared to EV surface-associated DNA, inner DNA displays a better quality and performance in NGS (Lazaro-Ibanez et al., 2019). Remarkably, compared to cfDNA, the reported average fragment length is longer in EVs. Thus, together with a nucleosome-associated pattern, fragments up to 4 kb are observed in exosome-derived DNA (Vagner et al., 2018; Lazaro-Ibanez et al., 2019; Malkin and Bratman, 2020) and chromosomal DNA has been found in large EVs such as oncosomes in prostate cells and in plasma of prostate cancer patients (Vagner et al., 2018).

Several works have reported that EV-shed DNA allows the detection of mutations that reliably reflects the mutational state in the tumor of origin (Kahlert et al., 2014; Thakur et al., 2014; Vagner et al., 2018; García-Romero et al., 2019; Garcia-Silva et al., 2019; Kunz et al., 2019). As a consequence of this, the use of EV-derived DNA for liquid biopsy purposes has begun to be exploited (Table 1). The first studies with plasma-derived EVs focused on the analysis of EGFR in lung cancer and glioblastoma (Figueroa et al., 2017; Krug et al., 2017; Castellanos-Rizaldos et al., 2018) and KRAS in pancreatic cancer (Allenson et al., 2017; Yang et al., 2017). More recently, predictive detection of BRAF mutation in circulating EV-DNA from pediatric central nervous system tumors has been described (García-Romero et al., 2019) together with the use of this mutation in seroma obtained post-lymphadenectomy in melanoma patients as a measure of minimal residual disease (Garcia-Silva et al., 2019). These and other studies demonstrate that the analysis of circulating EV-DNA can provide a prognostic value in diverse cancer types.

**Table 1:** Summary of the main reports performing DNA-EV analyses for liquid biopsy tests.

**References**	**Body fluid**	**Tumor type**	**Method**	**EV type**	**DNAase**	**Yield**	**DNA size**	**Biomarkers/platform**
Krug et al. (2017)	Plasma	NSCLC	Exo-Elution™ Plus extraction kit	Small and large EVs	No	ND	ND	EGFR T790M by NGS
Castellanos-Rizaldos et al. (2018)	Plasma	NSCLC	Exo-Elution™ Plus extraction kit	Small and large EVs	No	ND	ND	EGFR mutations by NGS
Allenson et al. (2017)	Plasma	Pancreatic cancer	Ultracentrifugation	Small EVs	No	ND	ND	KRAS mutations by ddPCR
Yang et al. (2017)	Serum	Pancreatic cancer	Ultracentrifugation	Small EVs	No	ND	ND	KRAS and TP53 mutations by ddPCR
San Lucas et al. (2016)	Pleural fluid	Pancreatic cancer	Ultracentrifugation	Small EVs	No	ND	>10kb	Tumor profiling by Illumina Hiseq2500
Figueroa et al. (2017)	CSF	Glioblastoma	Ultracentrifugation	Small EVs	Yes	ND	ND	EGFRvIII mutation by QPCR
Garcia-Silva et al. (2019)	Lymphatic exudate	Melanoma	Exo-Elution™ Plus extraction kit	Small and large EVs	No	ND	ND	BRAF V600E by QPCR
Hur et al. (2019)	BALF	NSCLC	Ultracentrifugation	Small EVs	No	ND	ND	EGFR mutations by QPCR
Song et al. (2019)	Pleural effusion fluid	NSCLC	Exo-Elution™ Plus extraction kit	Small and large EVs	No	ND	ND	Targeted-NGS
Lee et al. (2018)	Urine	Bladder cancer	ExoQuick-TC	Small EVs	No	ND	ND	Mutation analysis by target capture sequencing /CNV by whole genome sequencing

*CFS, cerebrospinal fluid; BALF, bronchoalveolar lavage fluid; NSCLC, non-small cell lung cancer; PDAC, pancreatic ductal adenocarcinoma; ND, not determined*.

## An Overview of cfDNA-Based Liquid Biopsies

Compared to EV-DNA, cfDNA has been much more extensively used in liquid biopsies. cfDNA is DNA freely circulating in the bloodstream. It is estimated that circulating tumor DNA might only be <0.01% of the total cfDNA (Pantel and Alix-Panabières, 2019). It is mainly originated during the apoptotic process, although senescence and necrosis also influence cfDNA release kinetics (Rostami et al., 2020). As a consequence of its origin, it is a highly fragmented nucleic acid.

cfDNA-based liquid biopsy can be used to detect specific tumor aberrations such as point mutations, loss of heterozygosity (LOH) or amplifications (Kerachian et al., 2019). These specific tumor genetic alterations (or a panel of them) are analyzed by targeting DNA sequencing techniques such as digital PCR (dPCR), beads–emulsion–amplification–magnetics (BEAMing), safe-sequencing system (Safe-SeqS), cancer personalized profiling by deep sequencing (CAPP-Seq), and tagged-amplicon deep sequencing (TAmSeq) (Heitzer et al., 2019). Alternatively, a non-targeted next-generation sequencing (NGS), commonly a whole-genome/exome sequencing can also be applied to cfDNA in order to extract information about copy number alterations (CNAs), DNA rearrangements or detection of subclonal mutations. The biggest drawback of this approach is the lower overall sensitivity (above 1–5%) and the need for higher concentrations of ctDNA whereas targeted DNA sequencing techniques can reach a limit of detection (LOD) minor than 0.01% (Pantel and Alix-Panabières, 2019). However, ultra-sensitive detection in plasma cfDNA samples can be achieved by analyzing hundreds to thousands of mutations previously identified by tumor genotyping (Wan et al., 2020) or by using genome-wide mutational integration (Zviran et al., 2020).

The most frequent application of cfDNA-based liquid biopsy is the monitoring of therapy response and the analysis of minimal residual disease (MRD) in clinical samples in order to anticipate the relapse of the disease before its detection with current clinical techniques (e.g., CT or PET/CT, multiparametric flow cytometry, immunohistochemistry) (Cheng et al., 2019; Pantel and Alix-Panabières, 2019). In colon cancer, the positive detection of mutations in cfDNA efficiently demonstrated prognostic value in a cohort of 230 colorectal cancer patients in which cfDNA positive status was associated to inferior recurrence-free survival (Tie et al., 2016). In breast cancer, the use of ddPCR on cfDNA samples to validate previously identified chromosome rearrangements reached a sensitivity of 93% and a specificity of 100% (Olsson et al., 2015). Targeted capture sequencing analysis of ctDNA defined MRD-associated mutations and even predict the genetic events of subsequent metastatic relapse more accurately than sequencing of the primary breast cancer (Garcia-Murillas et al., 2019). In a landmark study in lung cancer, Swanton and colleagues developed a NGS-based mutational panel of 12–30 SNVs, with addition of clinical and radiological evaluations. The detection of SNVs in ctDNA seemed to correlate with clinical evidence of NSCLC relapse with a ctDNA detection threshold <0.1% (Abbosh et al., 2017). These and other studies (Reinert et al., 2016; Chaudhuri et al., 2017; Schøler et al., 2017) demonstrate how targeted and genome-wide DNA sequencing techniques provide an accurate information during disease monitoring.

## cfDNA *VS*. EV-NA, Who is the Winner?

It should be first noticed that when defining blood-derived cfDNA it is unclear the physical state/source of it. It can be effectively circulating DNA, or circulating in association with protein complexes or even associated to EVs (Jahr et al., 2001; Thierry et al., 2016). Importantly, standard protocols for cfDNA isolation do not exclude EV-shed DNA although neither concentrate nor select for it as other approaches do.

**Figure 1:**
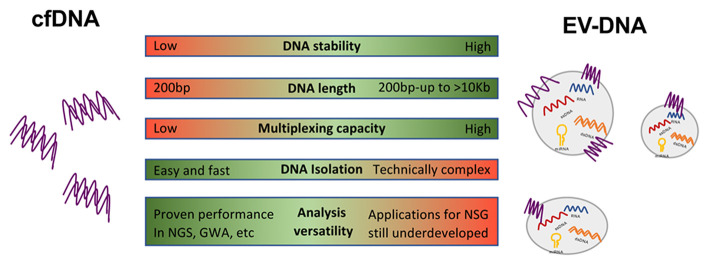
Advantages and disadvantages of EV-associated DNA and cfDNA. Circulating cell-free (cfDNA) shows a half-life of 2 h. However, extracellular vesicles (EVs) and associated content are stable for longer times. EVs display a wide range of DNA fragment sizes compared to highly fragmented cfDNA. Multiple EV cargo allows for polyvalent multiplexed analysis. DNA isolation is easy and fast for cfDNA but is technically more complex and time-consuming in the case of EVs. Non-targeted sequence-based analyses in EV-DNA are still underdeveloped while cfDNA has proven remarkable performance in next-generation sequencing techniques (NSG) and studies based in gene-wide analysis (GWA).

An overview of the differences between EV-DNA and cfDNA are summarized in Figure 1. EV-DNA is more stable than cfDNA whose half-life is estimated in <2 h (Cheng et al., 2016) forcing fast protocols of sample collection. On the contrary, EVs and their cargo show a considerable long-term stability in body fluids that facilitate their analysis in biobanked samples (Kalra et al., 2013). In addition, the fragmented nature of cfDNAs makes it difficult to generate a reliable genomic characterization for NGS, which in turn requires barcode and deep sequencing (Mouliere et al., 2011). On the other side, the presence of long EV-DNA fragments, that in cases display a chromatin structure favors amplification and better performance in NGS (Lazaro-Ibanez et al., 2019). Nevertheless, to date, most liquid biopsy studies focused on GWA and non-targeted sequencing have been performed in cfDNA such as methylation analyses (Sprang et al., 2020), DNA fragmentation patterns (Cristiano et al., 2019), or microbiome-DNA signatures (Poore et al., 2020) probably due to the delay in the application of those techniques to EV-based liquid biopsy.

Another advantage of exosomes and other EVs-based liquid biopsy is the possibility of multiplexing analyses of DNA with other EV cargo such as miRNA, long-non coding RNA, proteins, etc., that can provide a highly accurate information about the disease and will facilitate personalized and time-shaped medicine (LeBleu and Kalluri, 2020).

Few studies provide insights about the comparison of both cfDNA and EV-DNA in plasma. In a cohort of 84 non-small cell lung cancer patients, the combined use of exosomal-RNA and ctDNA sequencing improved the detection of EGFR (epidermal growth factor receptor)-activating mutations up to 98 vs. 84% for ctDNA alone (Krug et al., 2017). Remarkably, the best improvement was observed in the group of patients with intrathoracic metastatic disease known to have low levels of cfDNA. Moreover, the combined use of EV-RNA/DNA (EV-NA) together with cfDNA overcame the limited abundance of the EGFR T790M mutation and other EGFR mutations and obtained improved sensitivity and specificity than cfDNA alone (Castellanos-Rizaldos et al., 2018, 2019). These studies highlight the enhanced performance of combining EV-associated nucleic acids together with cfDNA vs. cfDNA analysis alone.

Following with the improved performance of EV-DNA tests, in a recent study, the assessment of Rhesus D genotype and gender by coupling quantitative PCR to EV-DNA and cfDNA isolation from maternal blood gave successful results with both approaches although only EV-DNA achieved a 100% of sensitivity and specificity for both assays (Yaşa et al., 2020).

An important drawback in EV-DNA tests is the lack of standardized isolation methods compared to cfDNA that implies additional steps of optimization, that are technically more complex and required clinical validation (Théry et al., 2006; Royo et al., 2020).

## There is Future Beyond Plasma! Use of Circulating EV in Other Biofluids

The secretion of EVs is a universal process and consequently EVs have been detected, isolated and analyzed in many biological fluids such as plasma, urine, cerebrospinal fluid (CSF) or bronchoalveolar lavage fluid (BALF) suggesting that these fluids can be explored for EV-based liquid biopsy tests (González and Falcón-Pérez, 2015; Gui et al., 2015; Broggi et al., 2019; Carnino et al., 2019; Kim et al., 2020). Until now, most of the studies based on other biofluids than blood have been focused on the quantification of exosomes or specific protein biomarkers. However, the analysis of mutations suggests that other fluids could be enriched in EV-associated DNA compared to plasma providing increase sensitivity. This is the case for lymphatic exudate also called exudative seroma in melanoma (Garcia-Silva et al., 2019). A combined approach isolating EV-associated nucleic acids together with cfDNA yield 600 times more copies of BRAFwt in this biofluid than in plasma. Thus, the limit of detection and sensitivity of the technique can be improved by choosing a fluid anatomically close to the tumor that would be enriched in tumor-derived EVs and consequently in EV-NA cargo. Another example of such an improvement is the analysis of CSF as an alternative to the scarce detection of biomarkers in blood for brain neoplasms. Thus, in glioblastoma patients, the use of CSF-derived EVs yield high specificity for the detection of tumor-associated EGFR amplifications (Figueroa et al., 2017). Additionally, profiling of BRAFV600E mutation in exudative seroma-derived EV-NA obtained after lymphadenectomy efficiently detected minimal residual disease and showed a strong prognostic value in a small cohort of stage III melanoma patients (Garcia-Silva et al., 2019). This study implies that malignancies such as breast cancer, melanoma, or others in which lymphadenectomy is performed could benefit from testing seroma-derived EV-NA for detection of minimal residual disease. It has been also proposed that NGS using EVs isolated from pleural fusions and urine could effectively replace tissue-based NGS in cases where there is a shortage of tissue (Lee et al., 2018; Song et al., 2019). Recent studies have demonstrated that EVs successfully isolated from BALF of lung cancer patients contain an abundant amount of dsDNA, and that liquid biopsy for EGFR genotyping using BALF is tissue-specific and extremely sensitive compared to cfDNA analysis (Hur et al., 2019; Kim et al., 2020). In summary, these works drive attention to the relevance of the selection of the ideal fluid as a source of EVs. The anatomical proximity to the tumor or disease and the intrinsic EV concentration certainly impact in the yield of the isolation process and consequently in the sensibility of the tests.

## Concluding Remarks

Compared to other types of liquid biopsy biomarkers, EV-DNA tests are in their early steps. However, they could provide additional analytical power to more established diagnostic methods due to the relatively abundant cargo of nucleic acids displaying increased stability compared to cfDNA. Although a few studies suggest that EV-NA-based tests could perform slightly better than cfDNA tests, more comparative analyses will be required to evaluate EV-NA capacity to overcome or complement cfDNA-based liquid biopsy. Combined EV-DNA/RNA with cfDNA approaches could amplify disease information and improved the specificity and the limit of detection in the tests, for example in the detection of some tumor mutations known to be underrepresented in cfDNA. On the other hand, there is a need for increased sensitivity that could boost non-invasive early detection and also enhance the analysis of minimal residual disease. Due to the heterogeneity of EV cargo, multiplexed assays combining DNA analysis with additional information on other biomolecules could also serve for a more personalized medicine. In addition, alternative biofluids are coming on the scene demonstrating improved performance than blood-based tests due to their enrichment in tumor-specific EVs. Finally, together with targeted-sequencing techniques, the development of diverse types of GWA and the capacities of machine learning to combine multiple layers of information, will definitively expand the applicability of EV-DNA-based liquid biopsy.

## Author Contributions

HP and SG-S conceived and designed the manuscript. SG-S, MG, and HP wrote the manuscript. All authors contributed to the article and approved the submitted version.

## Conflict of Interest

The authors declare that the research was conducted in the absence of any commercial or financial relationships that could be construed as a potential conflict of interest.
